# Supplementary material for: Changes in Hox Gene Chromatin Organization during Odontogenic Lineage Specification
Source: Genes (Basel). 2023 Jan 12;14(1):198. doi: 10.3390/genes14010198 (PMC9859321; doi:10.3390/genes14010198)
Supplement: Supplementary file 1 [file genes-14-00198-s001.zip › genes-2110007-supplementary.pdf]

Table S1

Primer pairs for Real-Time PCR

Gene	Forward Primer	Reverse Primer
mALP	AACAACCTGACTGACCCTTCG	AATCCTGCCTCCTTCCACC
mOCN	CAGGAGGGCAATAAGGTAGT	CGTAGATAGCGTTTGTAGGC
mRUNX2	TTCTCCAACCCACGAATGCAC	CAGGTACGTGTGGTAGTGAGT
mOSX	TGATGGGACAGAGTGAGCTG	CTGTCCCGTCTCCTCTCAAG
mCDX4	CGAGAAGACTGGAGCGTGTA	TGTAGTCGGTCGAGCAGAAA
mACTIN	GCTACAGCTTCACCACCACA	TCTCCAGGGAGGAAGAGGAT
hEZH2	CGAGAGTGTGACCCTGACCTC	CACATCAGACGGTGCCAGC
hACTIN	TTGCTGACAGGATGCAGAAG	GTACTTGCGCTCAGGAGGAG

Primer pairs for ChIP PCR

Gene	Forward Primer	Reverse Primer
hHOXA2 ChIP	TTACACTCGCGCACATTCAC	GGATGTCCCGGAGTACGTG
hHOXA3 ChIP	GTAAAACCTGGCCCAAAAGG	TTATGACCCTTTCCCCATCA
hHOXA4 ChIP	GTAAAACCTGGCCCAAAAGG	TTATGACCCTTTCCCCATCA
hHOXA9 ChIP	TGATGGCGTGATTAATTGTGA	GGCCAACGACGATTAAAAGA
hHOXA10 ChIP	GCCCTATAAAAGCCCATCCT	TAAGAAATCGCTGCAAAACC
hHOXB2 ChIP	GAAGGGGATGAGGAAAAGGT	AAACACCAGAGGACCACGAC
hHOXB3 ChIP	CACCCCTTGACTTTGTCTCC	TCTGACTTCTCGGCGATTTT
hHOXB9 ChIP	GCACATTGCATCAGCATAAAA	GCCTGATAAAGCGTCAGCTC
hHOXC11 ChIP	CTGAGCCACTAAGCCAAAGG	CATGCCTGCCTTTATTGTCA
hHOXD9 ChIP	GTCTCCAGCGCGCACTAT	AGCCTCCTCTCGGGTCAC

m=mouse; h=human

Figure S1

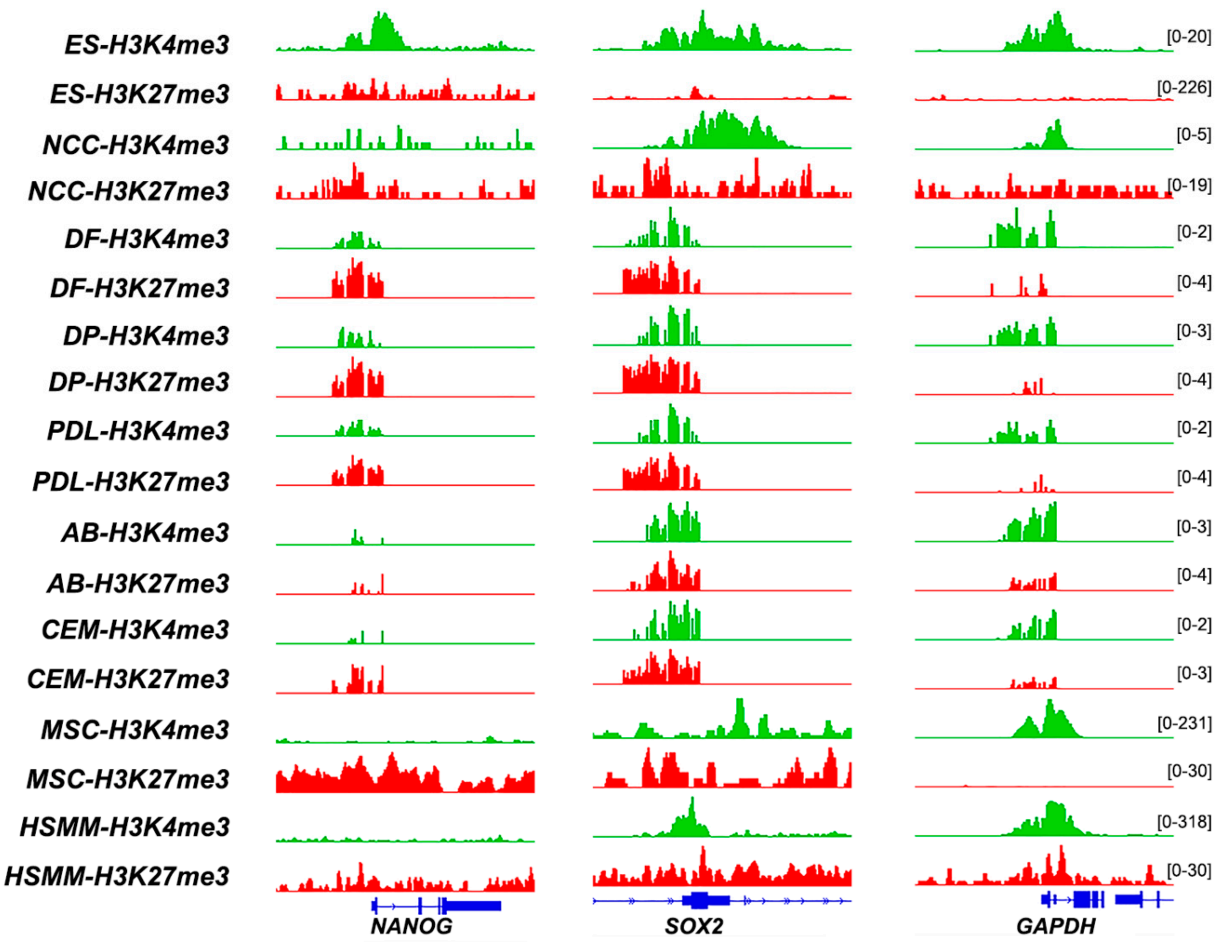
ES-H3K4me3
ES-H3K27me3
NCC-H3K4me3
NCC-H3K27me3
DF-H3K4me3
DF-H3K27me3
DP-H3K4me3
DP-H3K27me3
PDL-H3K4me3
PDL-H3K27me3
AB-H3K4me3
AB-H3K27me3
CEM-H3K4me3
CEM-H3K27me3
MSC-H3K4me3
MSC-H3K27me3
HSMM-H3K4me3
HSMM-H3K27me3
[0-20]
[0-226]
[0-5]
[0-19]
[0-2]
[0-4]
[0-3]
[0-4]
[0-2]
[0-4]
[0-3]
[0-4]
[0-2]
[0-3]
[0-231]
[0-30]
[0-318]
[0-30]
NANOG
SOX2
GAPDH

Figure S2

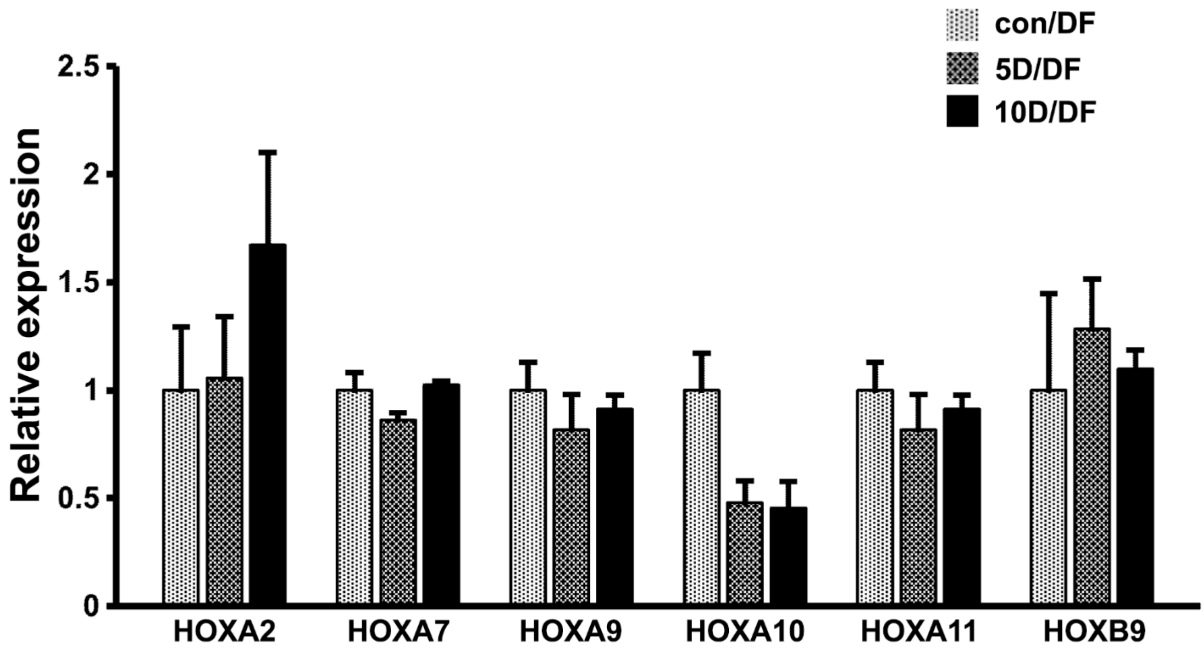

con/DF
5D/DF
10D/DF
Relative expression
2.5
2
1.5
1
0.5
0
HOXA2
HOXA7
HOXA9
HOXA10
HOXA11
HOXB9

Figure S3

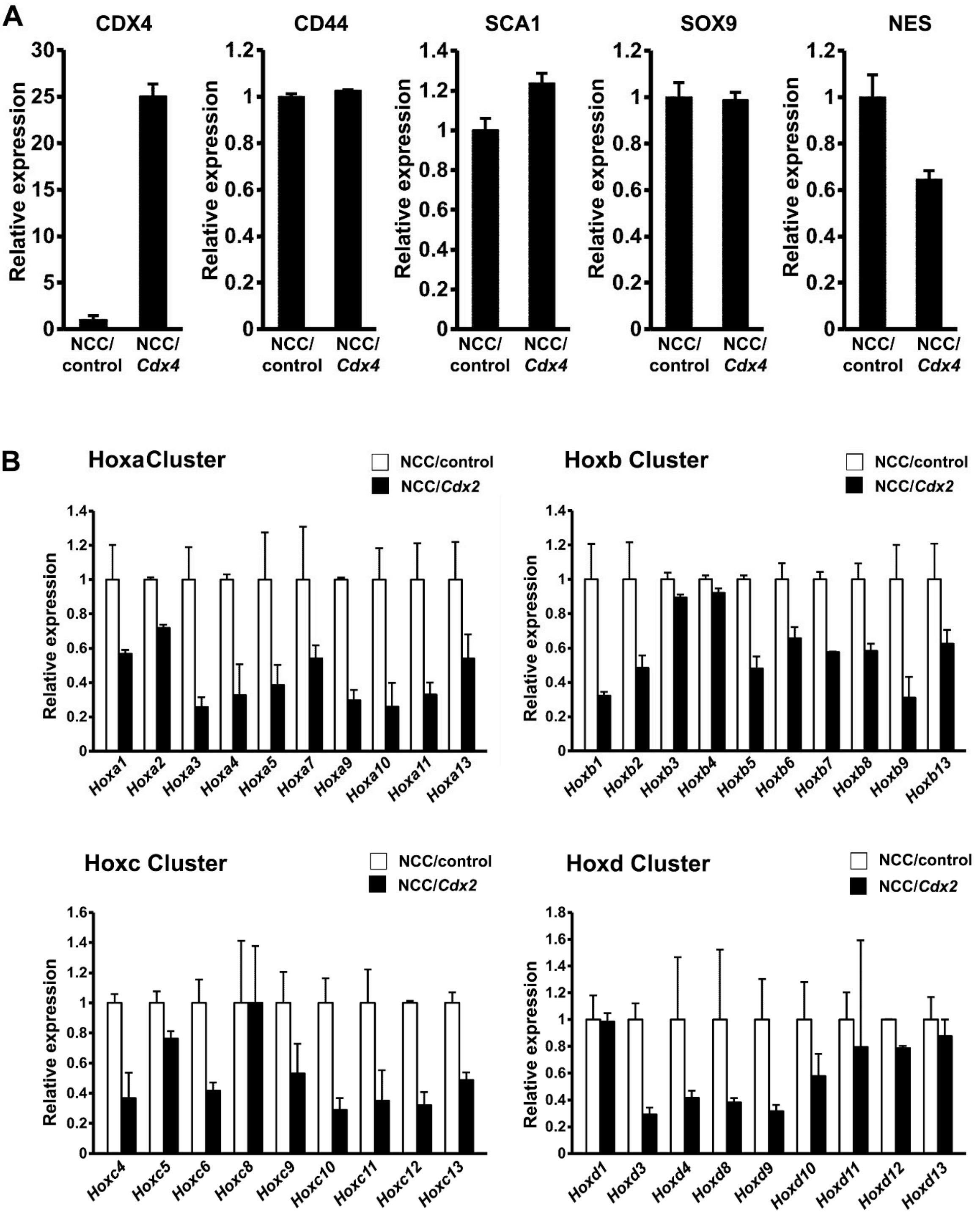

A
CDX4
CD44
SCA1
SOX9
NES
Relative expression
NCC/control
NCC/Cdx4
B
HoxaCluster
Hoxb Cluster
Hoxc Cluster
Hoxd Cluster
NCC/control
NCC/Cdx2
Relative expression
Hoxa1 Hoxa2 Hoxa3 Hoxa4 Hoxa5 Hoxa7 Hoxa9 Hoxa10 Hoxa11 Hoxa13
Hoxb1 Hoxb2 Hoxb3 Hoxb4 Hoxb5 Hoxb6 Hoxb7 Hoxb8 Hoxb9 Hoxb13
Hoxc4 Hoxc5 Hoxc6 Hoxc8 Hoxc9 Hoxc10 Hoxc11 Hoxc12 Hoxc13
Hoxd1 Hoxd3 Hoxd4 Hoxd8 Hoxd9 Hoxd10 Hoxd11 Hoxd12 Hoxd13
